# A new single nucleotide polymorphism affects the predisposition to thoracic ossification of the posterior longitudinal ligament

**DOI:** 10.1186/s13018-019-1481-6

**Published:** 2019-12-12

**Authors:** Peng Wang, Ze Teng, Xiaoguang Liu, Xiao Liu, Chao Kong, Shibao Lu

**Affiliations:** 1 Department of Orthopedics, Xuanwu Hospital of Capital Medical University, 45 Changchun Street, Xicheng, Beijing, 100053 People’s Republic of China; 2 Department of Radiology, Cancer Hospital Chinese Academy of Medical Sciences, Beijing, 100021 People’s Republic of China; 3 Department of Orthopedics, Peking University Third Hospital, Beijing, 100191 People’s Republic of China

**Keywords:** COL6A1, Thoracic, Ossification of the posterior longitudinal ligament

## Abstract

**Background:**

Thoracic ossification of the posterior longitudinal ligament (T-OPLL) is one of the common factors that cause thoracic spinal stenosis, which results in intractable myelopathy and radiculopathy. Our previous study first reported rs201153092A site mutation in the collagen 6A1 (*COL6A1*) gene as a potentially pathogenic locus for T-OPLL. We aimed to determine whether the rs201153092A site mutation causes abnormal expression of the *COL6A1* in Han Chinese patients with T-OPLL and whether this locus is also associated with cervical-OPLL.

**Methods:**

Peripheral blood was collected from a total of 60 patients with T-OPLL disease (30 patients carrying the rs201153092A site mutation in COL6A1 and 30 wild-type patients) and 400 northern Chinese individuals (200 cervical-OPLL patients and 200 control subjects) using the Sequenom system. The expression of COL6A1 was analyzed by enzyme-linked immunosorbent assay, reverse transcription-quantitative polymerase chain reaction, and Western blotting.

**Results:**

rs201153092A mutation resulted in markedly increased COL6A1 gene expression levels in peripheral blood samples. The allele frequency and genotype frequency results showed that this locus is no difference between cervical-OPLL patients and controls.

**Conclusions:**

The rs201153092A site mutation of COL6A1 can significantly increase the expression of COL6A1. The COL6A1 gene rs201153092A site polymorphism is a potential pathogenic mutation in T-OPLL disease, which may be only associated with the occurrence of T-OPLL.

## Background

Thoracic ossification of the posterior longitudinal ligament (T-OPLL) is characterized by pathological heterotopic ossification of this region. T-OPLL is a rare but has a high disability rate disease, which results in intractable myelopathy and radiculopathy, the prevalence of T-OPLL in individuals of Japanese ethnicity is 1.6–1.9%, and the mean age of onset is > 40 years old [1, 2]. T-OPLL has a marked ethnic predilection, as this disease occurs more frequently in Japanese and Chinese individuals. Although the pathogenesis of T-OPLL remains unclear and linked to several factors, including mechanical stress, genetic, and metabolic which probably contribute to the etiology of OPLL, most studies suggest that cervical-OPLL (C-OPLL) is a “genetic” disease [3–12]. Our previous whole-genome sequencing and candidate gene-association studies demonstrated that the presence of the rs201153092A single nucleotide polymorphism (SNP) in the *COL6A1* gene is potentially associated with T-OPLL susceptibility [13, 14]. Therefore, we hypothesize that *COL6A1* might be involved in the formation of OPLL of the thoracic spine.

*COL6A1* is a crucial component of the extracellular matrix and involved in membranous or endochondral ossification [15]. Although the *COL6A1* has been identified as potentially pathogenic loci for C-OPLL, the mutations reported in previous studies were located in the promoter regions or intronic regions of the *COL6A1* gene and lack relevant functional validation. The rs201153092A site mutation is located in the exonic region of the *COL6A1* gene. Mutation in the exonic region can affect the expression of the protein by affecting the amino acid sequence composition.

The present study aimed to determine whether the rs201153092A site mutation causes abnormal expression of the *COL6A1* gene in patients with T-OPLL among a Han Chinese population and to determine whether COL6A1 is involved in the pathogenicity of T-OPLL.

## Materials and methods

### Genotype-phenotype

This prospective study protocol was approved by the ethical committee for human subjects of the Peking University Third Hospital (Beijing, China). Informed consent was provided by all participating individuals. All participating individuals were enrolled in this study between May 2014 and December 2018. Diagnosis of OPLL was performed by orthopedic spine specialists based on clinical symptoms and computed tomography (CT) of the cervical and thoracic spine. The appearance of T-OPLL observed in CT was classified as segmental, continuous, mixed, or local disease type [16]. Furthermore, we collected patient age, gender, and neurological status data. Neurological status was evaluated by the Japanese Orthopedic Association (JOA) score for thoracic myelopathy (maximum 11 points). Inclusion criteria were northern Chinese Han patients with T-OPLL carrying the rs201153092A site mutation in COL6A1 and carrying the wild-type rs201153092G site. To determine whether the rs201153092A site mutation is also associated with cervical-OPLL or only associated with T-OPLL, we also enrolled C-OPLL patients for case-control association study. The required sample size for both groups in case-control association study was according to our previous research described in [14]. Type I error (*α* error = 5% by two-sided test) and power (1-β, 90%) were also defined. The sample size was calculated for each group. As a result, the sample size was estimated to be at least 185 patients for each group. Individuals who had lumbar spondylolisthesis, ankylosing spondylitis, diffuse idiopathic skeletal hyperostosis, and disc herniation of the thoracic spines were excluded in this study and did not take any drugs known to affect bone or calcium metabolism.

### Plasma COL6A1 enzyme-linked immunosorbent assay (ELISA)

Plasma collection and storage from all T-OPLL patients were performed using standard methods. Plasma COL6A1 levels were quantified using commercially available ELISA kits (Trust Specialty Zeal, Inc., San Francisco, CA, USA). All samples were assayed according to the manufacturer’s instructions and were run in duplicate. The optical density of each well was determined using a microplate reader at 450 nm. No interference and no cross-reactivity were expected based on the manufacturer’s instructions. All experiments were performed three times.

### Reverse transcription-quantitative polymerase chain reaction (RT-qPCR)

Total RNA was purified from all T-OPLL patient blood using the SK1321 RNA Blood Mini Kit (Sangon Biotech Co., Ltd., Shanghai, China). A one-column DNase digest (Sangon Biotech Co., Ltd.) was performed before the clean-up step to eliminate residual genomic DNA. cDNA was synthesized from total RNA (2 μg) using a RevertAid Premium Reverse Transcriptase kit (Thermo Fisher Scientific, Inc., Waltham, MA, USA). Relative qPCR was applied to quantify the mRNAs levels of COL6A1 using SYBR Green Real-Time PCR master mix on the LightCycler480 Real-Time System (Roche Diagnostics, Basel, Switzerland). All experiments were performed in triplicate and normalized to glyceraldehyde-3-phosphate dehydrogenase (GAPDH). Details of the primer sequences are listed in Table 1.

**Table 1:** Primer sequences used for quantitative polymerase chain reaction

Gene	Primer sequence
*COL6A1*	Forward 5′-CGAGATTGCCAAGGACTTCG-3′ Reverse 5′-AGGCTCTTGATGGCTTCCTT-3′
GAPDH	Forward 5′-TGGGTGTGAACCATGAGAAGT-3′ Reverse 5′-GAGTCCTTCCACGATACCAA-3′

#### Western blot (WB) analysis

Tissue lysates were obtained from all T-OPLL patients using ice-cold RIPA lysis buffer (Beyotime Institute of Biotechnology, Haimen, China) containing 100 mM PMSF as a protease inhibitor. Total protein (100 μg) was separated in a Bis-Tris polyacrylamide gel and transferred onto a nitrocellulose membrane. The membrane was then incubated in 1% bovine serum albumin containing primary rabbit anti-human polyclonal antibodies at 4 °C overnight. Following incubation with horseradish peroxidase-conjugated goat anti-rabbit antibody at room for 1 h, proteins were detected using electrochemiluminescence (EMD Millipore, Billerica, MA, USA). The following primary and secondary antibodies were used: anti-COL6A1 (1:2,000; cat. no. ab182744; Abcam, Cambridge, MA, USA) and goat anti-rabbit antibody (1,2,500; cat. no. CW0103M; Kangwei Biotech Co. Ltd). The blots were detected using a Kodak film developer (Fujifilm, Tokyo, Japan). Protein levels were quantified by densitometry analysis using Image-Pro Plus 6.0 software (Media Cybernetics, Inc., Rockville, MD, USA). Beta-actin was used as the endogenous control. All experiments were performed three times.

## Genotyping

EDTA-anticoagulated peripheral blood samples were obtained from all C-OPLL patients for DNA extraction. Genomic DNA samples were extracted from peripheral leukocytes with the standard procedure using a Wizard Genomic DNA Purification Kit (Promega Corporation, Madison, WI, USA). The polymerase chain reaction (PCR) fragments were submitted for Sanger sequencing at the Beijing Genomics Institute, and the forward and reverse sequence reads were assembled and analyzed in DNA Star version 7.1. Details of the primer sequences are listed in Table 2. The primer was used for PCR as described previously [17]. PCR was performed with 20 ng genomic DNA per 15-μl reaction mixture, containing 0.2 μM of each primer, 200 μM of deoxyribonucleotides, 50 mM KCl, 10 mM Tris HCl (pH 8.3), 1.5 mM MgCl2, and 0.5 units of Taq DNA polymerase in a DNA Gradient PCR machine (Bio-Rad Laboratories, Inc., Hercules, CA, USA). The thermocycling conditions were as follows: initial denaturation at 95 °C for 10 min; followed by 35 cycles of 95 °C for 30 s, annealing at an assay-specific temperature (48 to 65 °C) for 45 s, and elongation at 72 °C for 45 s; and a final terminal elongation step at 72 °C for 5 min. The PCR products were analyzed by direct sequencing using a BigDye Terminator v3.1 Cycle Sequencing Kit (Thermo Fisher Scientific, Inc., Waltham, MA, USA) with POP-7™ Polymer in a 3730XL DNA Analyzer with Sequencing Analysis Software version 5.2 (Thermo Fisher Scientific, Inc., Waltham, MA, USA).

**Table 2:** Details of the rs201153092 in COL6A1 and the associated primers

Gene	Primer sequence
*COL6A1*	Forward 5′-TGAAAGGGTGAGTGTCCAA-3′ Reverse 5′-GTGCCCAGTCCACTAAAGAG-3′

### Statistical analysis

All statistical analyses were performed using SPSS v17.0 software (SPSS, Inc., Chicago, IL, USA). Descriptive data for continuous variables are presented as the mean ± standard deviations. The Student’s *t* test was used to compare the means between 2 groups. The differences in T-OPLL subtypes between patients with or without COL6A1 gene mutation were applied using one-way analysis of variance with post hoc Fisher’s test. The genotypic and allelic distributions were obtained using the *χ*2 test. *P* ≤ 0.05 was considered to be statistically significant.

## Results

### Genotype-phenotype analysis

A total of 30 unrelated northern Chinese Han patients with T-OPLL carrying the rs201153092A site mutation in COL6A1 (17 men, mean age 56.35 ± 10.72 years; 13 women, mean age 53.31 ± 7.67 years) and 30 unrelated northern Chinese Han patients with T-OPLL carrying the wild-type rs201153092G site (16 men, mean age 56.75 ± 8.95 years; 14 women, mean age 50.71 ± 7.06 years) were enrolled in this study. No differences were found between these two groups in terms of sex, age, and JOA score at diagnosis. Phenotype-genotype associations were analyzed among the T-OPLL patients with or without the rs201153092A mutation (*n* = 30 per group). Additionally, radiological analysis of T-OPLL morphology revealed that the mutation-positive patients and mutation-negative patients exhibited no difference in the disease type classification (Table 3). A total of 200 unrelated northern Chinese Han C-OPLL patients with myelopathy and/or neurological dysfunction [97 men (mean age, 50.65 ± 6.38 years) and 103 women (mean age, 51.65 ± 7.01 years)] and 200 sex-matched unrelated healthy controls [100 men (mean age, 49.84 ± 6.46 years) and 100 women (mean age, 53.36 ± 5.63 years)] were enrolled.

**Table 3:** Clinical features of T-OPLL patients with or without rs201153092A mutation

Variable	rs201153092A (*n* = 30)	rs201153092G (*n* = 30)	*P*
Age (years)	55.03 ± 9.50	53.93 ± 8.55	NS
Male/female	17/13	16/14	NS
Continuous	8 (26.7%)	9 (30.0%)	NS
Local	2 (6.7%)	1 (3.3%)	NS
Segmental	6 (20.0%)	8 (26.7%)	NS
Mixed	14 (46.7%)	12 (40.0%)	NS
JOA score	3.45 ± 0.98	3.88 ± 0.78	NS

Data are presented as the means ± standard deviation or *n* (%). *T-OPLL*, thoracic ossified posterior longitudinal ligament; *NS*, not significant; *JOA score*, Japanese Orthopedic Association scoring system for thoracic myelopathy (maximum 11 points)

#### Analysis of COL6A1 levels in the blood of patients with T-OPLL

**Fig. 1:**
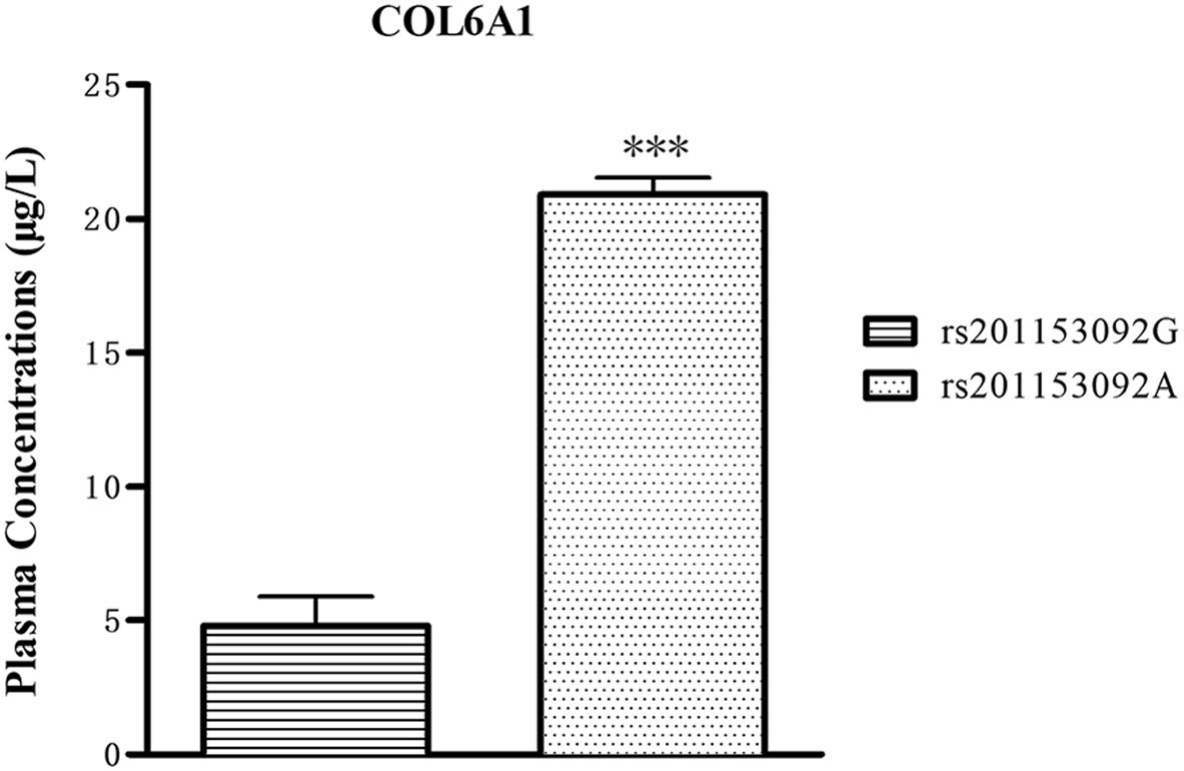
Plasma COL6A1 ELISA. The plasma COL6A1 level of T-OPLL patients with rs201153092A mutation was significantly higher than T-OPLL patients carrying the rs201153092G site. ***P < 0.001

**Fig. 2:**
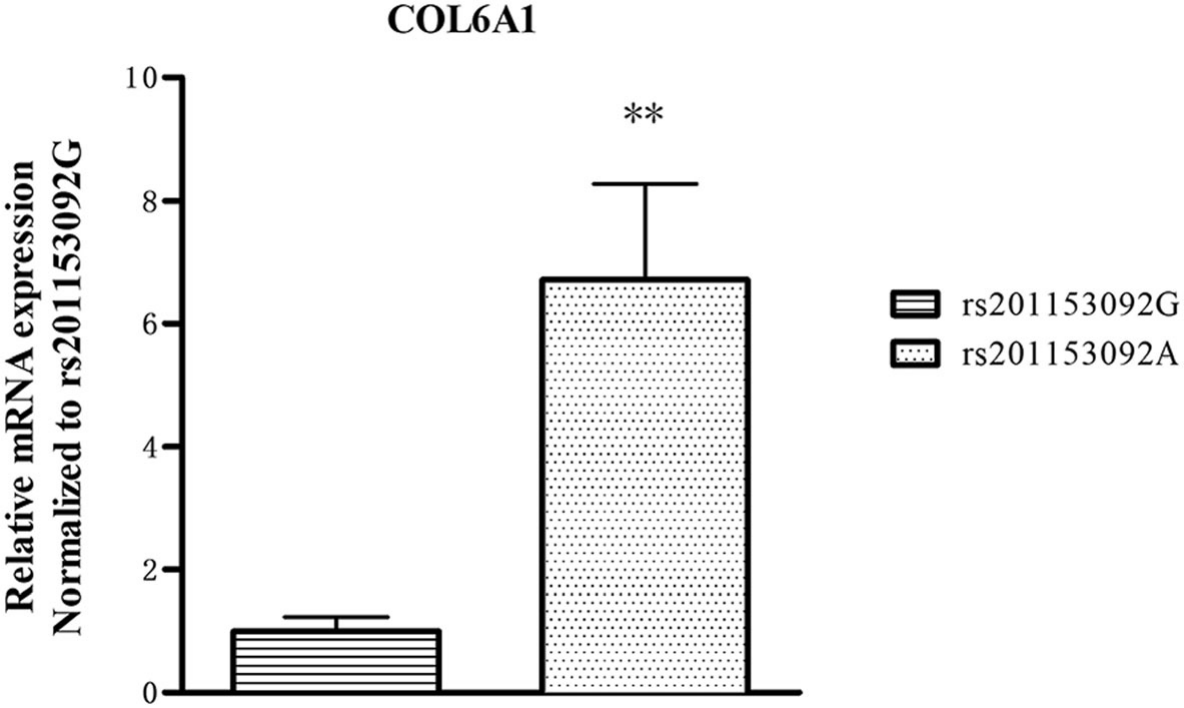
Analysis of COL6A1 mRNA expression. The mRNA expression levels of COL6A1 in T-OPLL patients with rs201153092A mutation were significantly higher than that those T-OPLL patients carrying rs201153092G. ***P < 0.001

The plasma concentration of COL6A1 was shown in Fig. 1; plasma COL6A1 concentration was significantly higher (~ 3-fold higher) in T-OPLL patients with rs201153092A mutation (20.90 ± 0.64 μg/l) compared with T-OPLL patients carrying the wild-type rs201153092G variant (4.79 ± 1.1 μg/l, *P* < 0.001). RT-qPCR analysis was performed using RNA extracted from peripheral blood cells (Fig. 2) and demonstrated that COL6A1 mRNA levels were ~ 6-fold higher in T-OPLL patients carrying the rs201153092A mutation than T-OPLL patients with the wild-type rs201153092G site (*P* < 0.01). Compared with wild-type T-OPLL patients, the rs201153092A mutation significantly increased COL6A1 gene expression, suggesting that this is a potential pathogenic locus that alters COL6A1 gene expression in cells.

#### COL6A1 protein expression in T-OPLL patients with rs201153092A mutation

**Fig. 3:**
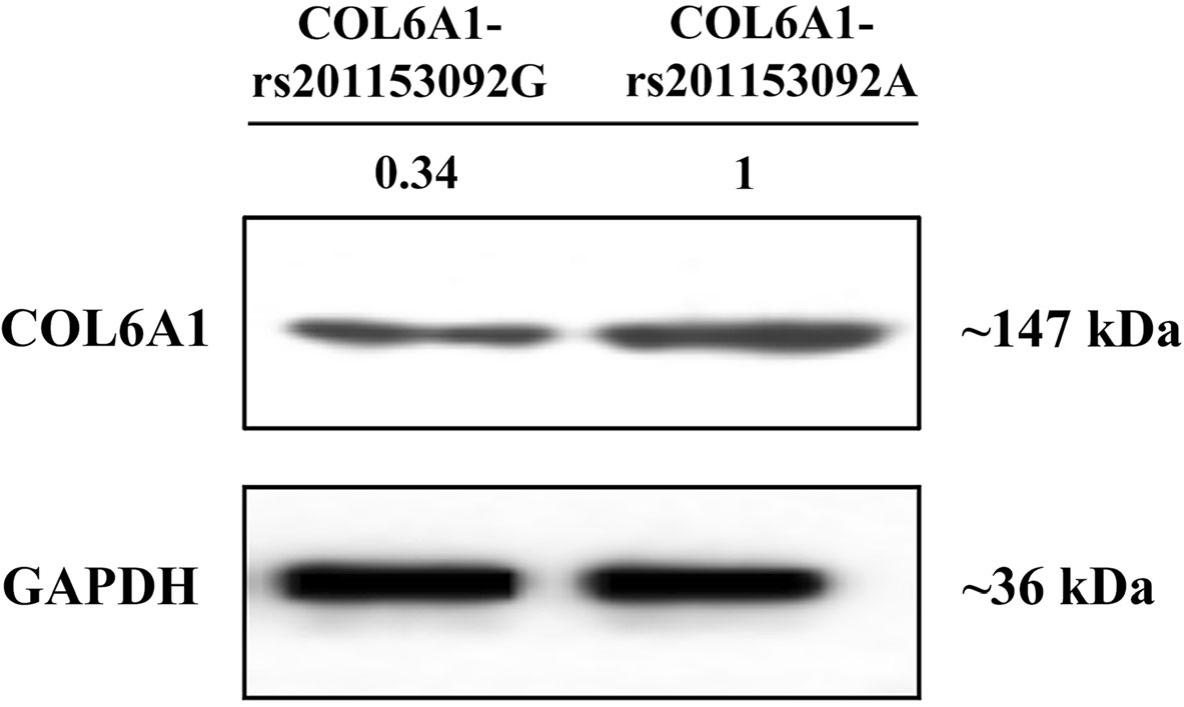
Protein expression of COL6A1. The expression levels of COL6A1 proteins detected by Western blot analysis

WB analysis revealed that the expression of COL6A1 protein was higher in T-OPLL patients with the COL6A1 gene rs201153092A mutation than T-OPLL patients carrying the wild-type rs201153092G variant (Fig. 3).

#### Association study

The allelic frequencies and genotype distributions of the rs201153092 are shown in Tables 4 and 5. No significant difference was observed in the genotype or allele frequencies of rs201153092 between cervical-OPLL cases and controls.

**Table 4:** Allelic frequencies of the rs201153092

SNP ID	*N*	Allele frequency (%)		*P* value
		Major allele	Minor allele	
rs201153092		G	A	
case	200	99.50	0.50	0.1568
control	200	100.00	0.00	

*N* number of subjects

**Table 5:** Genotype frequencies of the rs201153092

SNP ID	*N*	Genotype frequency (%)			Versus control	
		MM	Mm	mm	*P* value^a^	*P* value^b^
rs201153092		GG	GA	AA	–	0.1563
Case	200	198	2	0		
Control	200	200	0	0		

*N* number of subjects

^a^Dominant model: (major allele homozygote + heterozygote)/minor allele homozygote

^b^Recessive model: major allele homozygote/(minor allele homozygote + heterozygote)

## Discussion

Although the exact cause of the T-OPLL disease is still unclear, it is generally believed that genetic factors have an important role in the development of the disease. Some scholars believe that the accumulation of harmful missense mutations in the human genome creates a genetic basis for various complex diseases [18]. Disease progression is also affected by the gene expression in peripheral blood cells, with several genes reported to exhibit higher expression in the peripheral blood of OPLL patients compared with healthy controls [12, 19].

In this study, peripheral blood of T-OPLL patients with or without rs201153092A mutation was collected and analyzed to assess the role of the mutated gene locus. The results of ELISA and qPCR analysis demonstrated that the expression of *COL6A1* was significantly higher in the peripheral blood of T-OPLL patients carrying the rs201153092A mutation compared with patients without the mutation. WB were performed to determine *COL6A1* expression in patients with T-OPLL, and the results demonstrated that the expression of *COL6A1* protein was significantly higher in the T-OPLL patients with the mutation compared with those with the wild-type. Therefore, the rs201153092A site mutation may lead to overexpression of the *COL6A1*. We also showed that there was no difference in the OPLL classification and JOA score between T-OPLL patients with and without the rs201153092A mutation. The possible association between the rs201153092A site mutation in *COL6A1* and the severity of the T-OPLL phenotypes requires larger scale studies in the future.

The rs201153092 site of the *COL6A1* gene is located in the exonic region. The sequence of COL6A1 mRNA exhibits no difference between the wild-type and mutant. Thus, RT-qPCR and the Western blot analysis cannot distinguish between the wild-type and mutant *COL6A1*. The mutation in the exonic region enhances the expression level of the *COL6A1* gene mRNA which further translates more COL6A1 protein.

COL6 is a remarkable component of the extracellular matrix of many tissues including muscle, tendon, and cartilage [20]. Disorders caused by mutations of *COL6A1* genes mainly affect ligaments and muscles; mutations of the *COL6A1* polypeptide chains are causative of a broad spectrum of diseases in humans, including Ullrich congenital muscular dystrophy and C-OPLL [21]. Tanaka et al. [15] identified that molecular variants of the extracellular proteins may be implicated in the ectopic ossification observed in OPLL, and *COL6A1* gene may lead to increased bone mass. *COL6A1* gene which promotes bone formation is regulated by various pathways. Izu et al. [22] demonstrated that cell-cell interactions play an essential role in bone formation; *COL6A1* regulates bone formation through establishing communication cell networks at bone-forming sites. Cheng et al. [23] indicated that *COL6A1* gene exerts its biological functions through the Akt/PI3K pathway. However, the mechanism by which *COL6A1* gene facilitates T-OPLL remains unclear and we will try to explore it in the future and request additional experiments for this reason.

The results of this study demonstrated that the expression of *COL6A1* in T-OPLL patients carrying an rs201153092A mutation was significantly higher in peripheral blood and tissues than in patients without the mutation. It is suggested that the rs201153092A site mutation can lead to overexpression of *COL6A1* and play a role in the development of T-OPLL. To the best of our knowledge, there are currently no reports available on the rs201153092A locus related to C-OPLL; to determine whether the rs201153092A site mutation is also associated with cervical-OPLL or only associated with T-OPLL, we performed a case-control association study. And the result showed that this locus was not related to cervical-OPLL. However, this study lacks in-depth research and discussion on the mechanism by which *COL6A1* facilitates T-OPLL. Besides, due to the prevalence of T-OPLL, the disease is very rare; this study, T-OPLL patient sample size is small. Further studies with more participants of other ethnicities are needed to confirm these positive findings.

## Conclusions

In conclusion, the findings of this study suggest that the rs201153092A site mutation can lead to overexpression of COL6A1 and is a potential pathogenic mutation associated with T-OPLL. The results provide a potential basis for the pathogenesis of T-OPLL and the pathogenic role of COL6A1 in T-OPLL disease.

Further genetic studies with more participants are required to validate these findings and in appropriate model systems.

## Data Availability

All data generated or analyzed during this study are included in this published article.

## Abbreviations

COL6A1: Collagen 6A1
C-OPLL: Cervical ossification of the posterior longitudinal ligament
CT: Computed tomography
ELISA: Enzyme-linked immunosorbent assay
IHC: Immunohistochemical
JOA: Japanese Orthopedic Association
RT-qPCR: Reverse transcription-quantitative polymerase chain reaction
T-OPLL: Thoracic ossification of the posterior longitudinal ligament
WB: Western blot
